# Establishment of an orthotopic transplantation tumor model in nude mice using a drug-resistant human ovarian cancer cell line with a high expression of c-Kit

**DOI:** 10.3892/ol.2014.2537

**Published:** 2014-09-15

**Authors:** CUNJIAN YI, LEI ZHANG, LI LI, XIANGQIONG LIU, SHENGRONG LING, FAYUN ZHANG, WEI LIANG

**Affiliations:** 1 Department of Gynecology, The First Affiliated Hospital of Yangtze University, Jingzhou, Hubei 434000, P.R. China; 2 The Institute of Biophysics, Chinese Academy of Sciences, Beijing 100101, P.R. China

**Keywords:** c-Kit, cisplatin-resistant ovarian cancer, nude mice, orthotopic transplantation

## Abstract

The resistance of ovarian cancer to platinum-based chemotherapy is a critical issue in the clinical setting. The present study aimed to establish animal models to replicate this clinical condition, as well as to investigate the resistance mechanisms of ovarian cancer. A cisplatin (DDP)-resistant human ovarian cancer cell line, SKOV3/DDP, was screened, validated and injected subcutaneously into the neck of female nude mice. Following tumor establishment, the tumor was collected and cut into small sections, which were subsequently implanted into the ovaries of other nude mice. The growth of the orthotopic tumors was observed and the tumor-bearing mice were sacrificed and dissected. The orthotopic and metastatic tumor tissues were collected, sectioned, stained with hematoxylin and eosin and analyzed. In the present study, 16 nude mice underwent orthotopic transplantation surgery and a tumor model was successfully established in 14/16 of the mice, with an *in situ* tumor formation rate of 87.5%. Following euthanasia, a laparotomy demonstrated the tumor formation at the site of transplantation, as well as varying degrees of metastasis to additional organs and tissues. Therefore, the present study successfully established an orthotopic tumor transplantation model in nude mice using a c-Kit-positive DDP-resistant human ovarian cancer cell line. This model may represent a useful tool for investigating the resistance mechanism of ovarian cancer, as well as evaluating the efficacy of therapeutic strategies.

## Introduction

Ovarian cancer is highly malignant among gynecological tumors and is associated with an insidious onset, rapid progression and a complex early diagnosis, which leads to a poor prognosis and high mortality rates. Chemotherapeutic agents are an important means of adjuvant therapy for the treatment of patients with ovarian cancer. For example, combination therapy using cisplatin (DDP) and paclitaxel is considered to be the gold standard of chemotherapy (1). However, during clinical treatment, the majority of patients develop a drug resistance (2), which manifests as a tolerance to the chemotherapeutic agents or recurrence following treatment, leading to chemotherapy failure and a marked limitation of this treatment modality (3).

Previous clinical studies have shown that malignant ovarian cancer cells express the proto-oncogene, c-Kit and that the prognosis of patients exhibiting positive c-Kit gene expression is usually poorer. Schmandt *et al* (4) demonstrated that c-Kit was highly expressed in malignant ovarian tumors via immunohistochemistry, and indicated that c-Kit expression was associated with the histological tumor grade. The present study used a DDP-resistant cell line with a high expression of c-Kit to establish an orthotopic transplantation animal model, stimulating human ovarian cancer with regard to onset, location, mechanism and histological and biological characteristics, was used to investigate association between c-Kit gene expression and drug resistance and degree of malignancy in ovarian cancer.

## Materials and methods

### Experimental cells

The SKOV3 human ovarian cancer cell line and its DDP-resistant variant, SKOV3/DDP were purchased from the Tumor Cell Bank of the Chinese Academy of Medical Sciences (Beijing, China).

### Animals

BALB/c nude mice (weight, 18–20 g) were purchased from the Model Animal Research Center of Nanjing University (Nanjing, China). The mice were maintained in the animal facility at the Institute of Biophysics, Chinese Academy of Sciences (Beijing, China) under specific pathogen-free (SPF) conditions and housed in plastic cages in groups of six in Animal Resource Service facilities. The study was approved by the Institution of Animal Care and Use Committee at the Institute of Biophysics, Chinese Academy of Sciences (SYXK2013-02).

### Experimental therapeutic agents and reagents

Broad-spectrum antibiotic ampicillin sodium powder (5 mg/bottle; North China Pharmaceutical Co., Ltd., Shijiazhuang, China) and surgical anesthetic sodium pentobarbital solution (10 mg/ml) were administered to the nude mice following sterilization via filtering through a 0.22-μm filter membrane.

### Experimental instruments

A surgical dissection microscope (Nikon SMA800; Nikon Corporation, Tokyo, Japan) was used to analyze the microscopic morphology.

### Cell culture and validation

SKOV3 and SKOV3/DDP cells were cultured in RPMI-1640 medium containing 10% fetal bovine serum. The cells were collected in the logarithmic growth phase using centrifugation at 86 × g (TDZ5-WS multicarrier auto-balancing centrifuge; Hunan XiangYi Centrifuge Instrument Co., Ltd., Changsha, China), counted and resuspended in fresh medium. MTT assay (Beijing Solarbio Science & Technology Co., Ltd., Beijing, China) and quantitative polymerase chain reaction (qPCR) methods were used to validate the DDP resistance and c-Kit gene expression in the SKOV3/DDP cells.

### Establishment of the animal model

Four nude mice were used for the subcutaneous tumor model. Cultured SKOV3/DDP cells in the logarithmic growth phase were harvested, prepared into a cell suspension at a cell density of 2×10^7^ cells/ml and injected subcutaneously into the neck of the nude mice (0.2 ml/mouse). The mice were returned to their cages for continuous feeding following confirmation that there was no leakage at the injection site. The size of the subcutaneous tumor was regularly measured using a vernier caliper to monitor its growth. The tumors were excised and used for the orthotopic implantation experiment when the diameter was >1 cm.

Six-week-old SPF-level nude mice (weight, 17–18 g) were used for the orthotopic transplantation model. The animals were allowed to adapt to the experimental environment for one week. The mice with subcutaneous tumors were sacrificed via cervical dislocation and the tumors in the neck were collected. Following the removal of the capsule and connective tissues, the tumor was cut into small sections (~1 mm^3^), which were placed in ice-cold phosphate-buffered saline for further investigation. The recipient nude mouse was anesthetized with sodium pentobarbital and fixed on the operating table. The abdominal cavity of the mouse was opened to expose the ovaries, which were cut under the Nikon SMA800 dissecting microscope (Nikon Corporation). The prepared tumor sections were directly implanted in the right ovary and sutured using an 8–0 absorbable suture. The abdomen was closed layer-by-layer using sterile silk. Post surgery, the mouse was placed on a warm pad, administered with anti-infection treatment and maintained under SPF conditions.

Following orthotopic transplantation, the food uptake, physical activities and mental conditions of the model nude mice were monitored daily. Weight measurement and abdominal palpation were performed every third day. When the abdominal mass was obviously palpable, the tumor-bearing mice were sacrificed and dissected to observe the *in situ* tumor growth, as well as tumor infiltration and metastasis to other tissues and organs. The tumor mass at the orthotopic transplantation site, as well as other associated tissues, were dissected, sectioned, stained with hematoxylin and eosin (H&E) and analyzed under a microscope (SCN400; Leica, Mannheim, Germany).

### Statistical Analysis

Data are presented as the mean ± standard deviation (n=5) for individual experiments, unless noted otherwise. Differences between the variables of groups were compared using Student’s t-test. Statistical analysis was performed using SPSS 11.0 (SPSS, Inc., Chicago, IL, USA). P<0.05 was considered to indicate a statistically significant difference for MTT. However, for qPCR, P<0.01 was considered to idicate a statistically significant difference.

## Results

### Cell culture and verification

**Figure 1:**
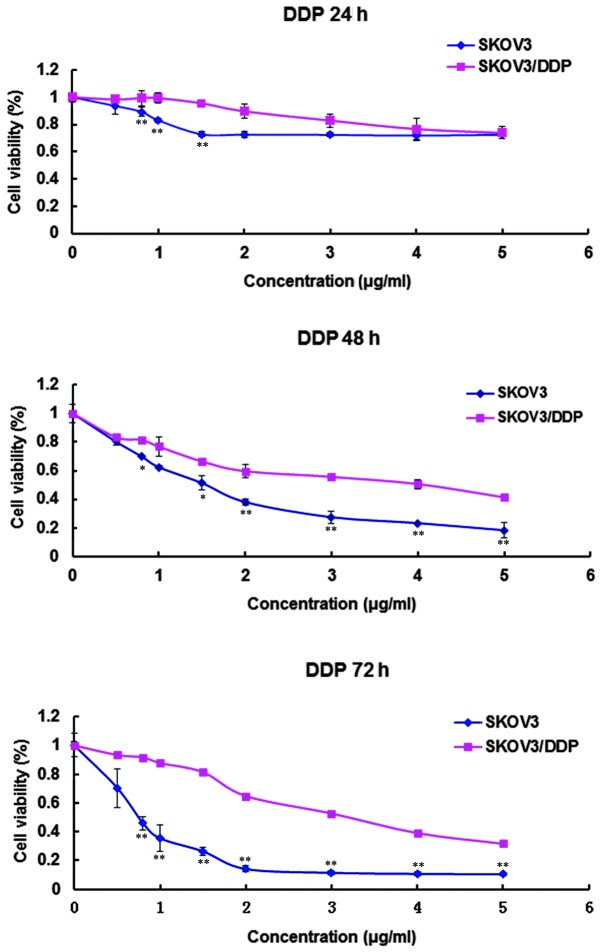
Time- and dose-dependent cytotoxic effect of cisplatin (DDP) on SKOV3 and SKOV3/DDP cells determined using an MTT method. ^*^P<0.05 vs. SKOV3 cells, ^**^P<0.01 vs. SKOV3 cells.

**Figure 2:**
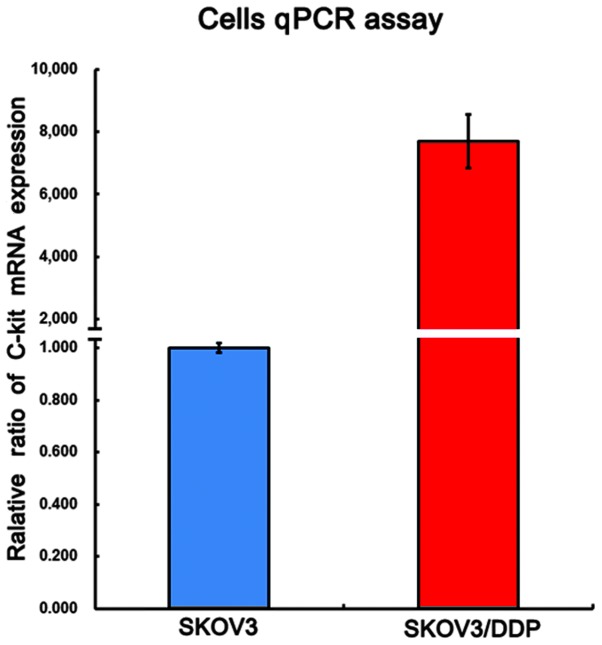
qPCR analysis of c-Kit mRNA expression in SKOV3 and SKOV3/DDP cells. qPCR, quantitative polymerase chain reaction analysis; DDP, cisplatin. ^**^P<0.01 vs. SKOV3 group.

The dose- and time-dependent cytotoxic effects of DDP on the cultured SKOV3 and SKOV3/DDP cells were determined using an MTT colorimetric method. Cell growth and survival curves were then plotted (Fig. 1). As shown in Fig. 1, DDP resistance was significantly higher in the SKOV3/DDP cells compared with the SKOV3 cells following treatment with DDP for 24, 48 and 72 h. Furthermore, qPCR analysis (Fig. 2) revealed that c-Kit mRNA was expressed in the SKOV3 and SKOV3/DDP cells, and that the expression of c-Kit in the DDP-resistant SKOV3/DDP cells was significantly higher compared with that in the non-resistant SKOV3 cells.

### Growth and metastasis of tumors transplanted orthotopically

A total of 16 nude mice were used to establish an orthotopic tumor transplantation model with SKOV3/DDP ovarian cancer cells. Two mice succumbed within one week of surgery; however, the orthotopic tumor transplantation model was successfully established in the remaining 14 mice, with a success rate of 87.5%. Table I shows the growth and metastasis findings of the tumor that was transplanted orthotopically *in vivo*.

**Figure 3:**
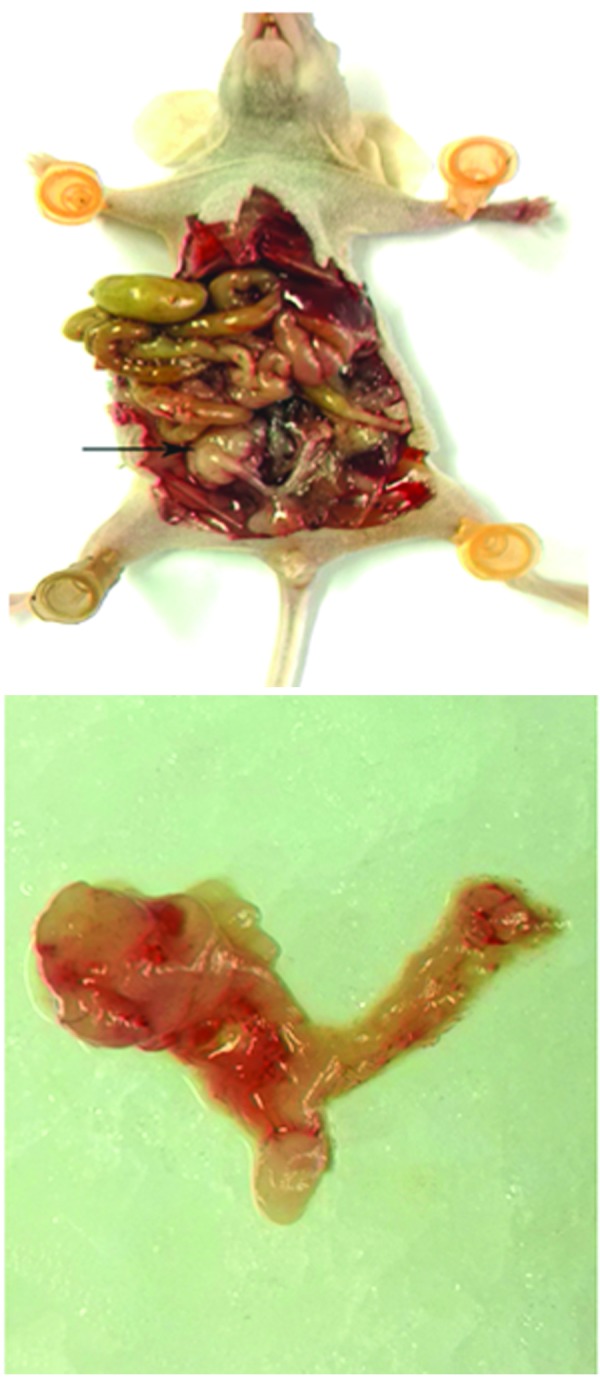
Anatomic images of an orthotopically-transplanted ovarian tumor in a nude mouse model.

**Figure 4:**
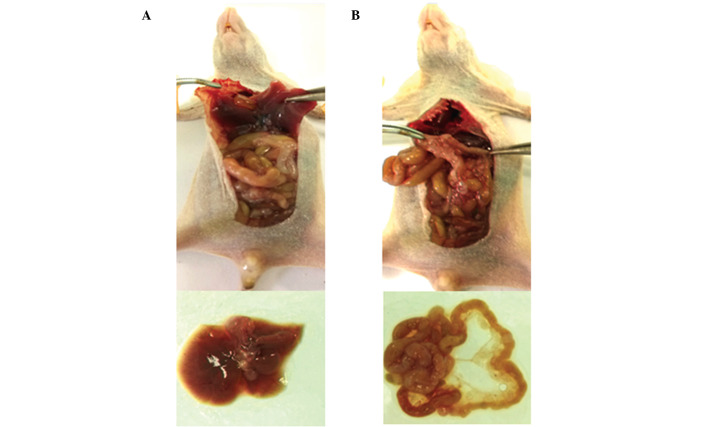
Anatomic images showing ovarian cancer metastases to the (A) liver and (B) mesentery in an orthotopic transplantation nude mouse model.

Abdominal swellings gradually appeared in the nude mice ~8–10 weeks after the successful orthotopic tumor transplantation. In addition, the mice exhibited loss of body weight and appetite, accompanied by decreased activity levels. Abdominal palpation revealed a palpable mass with poor mobility. The tumor-bearing mice were sacrificed via cervical dislocation when the abdominal mass was >2 cm in diameter. A laparotomy identified the formation of a relatively large mass in the transplantation side (Fig. 3), with a hard texture and varying degrees of adhesion to the surrounding tissues. A small quantity of dark-red intraperitoneal fluid was also observed. In certain mice, metastatic lesions were found in the abdominal organs, including the liver and mesentery (Fig. 4).

### Pathology

**Figure 5:**
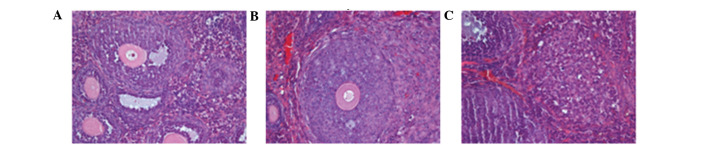
Anatomic images showing (A) the healthy ovaries in a control mouse and (B and C) the transplanted and metastatic ovarian cancer tumors in the ipsilateral and contralateral sides in an orthotopic transplantation nude mouse model. Hematoxylin and eosin stain; magnification ×400.

**Figure 6:**
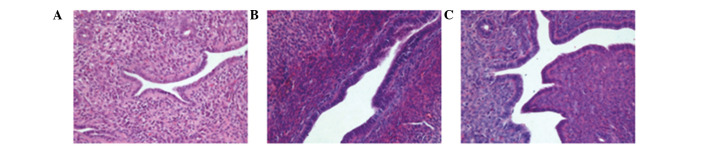
Microscopic images of (A) a pathological section of a healthy fallopian tube in a control mouse and (B and C) metastatic tumors in the bilateral fallopian tubes in an orthotopic transplantation nude mouse model. Hematoxylin and eosin stain; magnification, ×400.

The orthotopic tumors and organs with suspected metastatic tumors were resectioned, stained with H&E and analyzed under a microscope. A large number of ovarian cancer cells were observed in the bilateral ovaries and demonstrated infiltrative growth. The cell morphology was observed to be the same as that of the parental subcutaneous tumor, with large dark-stained nuclei and a high karyoplasmic ratio. The cells exhibited evident atypia, and a dense and disordered arrangement with visible interstitial cells (Fig. 5). Certain mice exhibited visible bilateral tubal metastasis of the tumor cells, with a morphology the same as that of the tumor cells that were transplanted orthotopically (Fig. 6). In the liver, the hepatic cord cells showed disarrangement, with evident inflammatory cell infiltration and small nodule formation. There were no metastatic tumor cells visible in the uterine tissue.

## Discussion

Resistance to platinum-based chemotherapy in ovarian cancer is a critical issue in the clinical setting. In a previous clinical study, c-Kit gene expression was identified to be closely associated with drug resistance and malignancy in ovarian cancer (5), which was consistent with previous findings (4). In the preliminary experiments, using 4T1-LUC cells, an orthotopic transplantation model of ovarian cancer was established. The implanted ovarian tumor model exhibited biological characteristics, and a metastatic rate and pattern that were closest to the clinical state. To understand the association between c-Kit gene expression, and drug resistance and malignancy in ovarian cancer, an animal model that simulates human ovarian developmen cancer in terms of onset, location, mechanism and histological and biological characteristics was required.

In the present study, SKOV3 and SKOV3/DDP human ovarian cancer cell lines were used and their sensitivity to DDP and c-Kit expression was assessed via MTT assay and qPCR. The c-Kit gene was identified to be expressed in the two cell lines and was significantly highly expressed in the DDP-resistant SKOV3/DDP cells, which correlated with its drug resistance. Using the SKOV3/DDP cells, an orthotopic tumor transplantation model was successfully established in the nude mice that closely simulated the occurrence, development and metastasis of human ovarian cancer. This model may facilitate further investigations into the association between c-Kit gene expression and malignancy in ovarian tumors. Furthermore, the model may provide a valuable tool for investigating the underyling mechanism of drug resistance in ovarian cancer, as well as the late-phase evaluation of the *in vivo* efficacy of novel treatment plans.

## Figures and Tables

**Table I:** Organs with metastatic tumors in the mouse model.

Mouse no.	Organs with metastatic tumors
1	Bilateral ovaries, fallopian tubes and mesentery
2	Bilateral ovaries and fallopian tubes
3	Bilateral ovaries, fallopian tubes and omentum
4	Bilateral ovaries, fallopian tubes and mesentery
5	Bilateral ovaries, fallopian tubes, mesentery and hepatic surface, with small number of bloody ascites
6	Bilateral ovaries, fallopian tubes and liver
7	Bilateral ovaries, fallopian tubes, mesentery and intestinal surface
8	Bilateral ovaries, fallopian tubes, mesentery and omentum
9	Bilateral ovaries, fallopian tubes, mesentery and hepatic surface, with small number of bloody ascites
10	Bilateral ovaries, fallopian tubes and hepatic surface
11	Bilateral ovaries and fallopian tubes
12	Bilateral ovaries
13	Bilateral ovaries
14	Ipsilateral ovary
